# Expanding horizons of tandem repeats in biology and medicine: Why ‘genomic dark matter’ matters

**DOI:** 10.1042/ETLS20230075

**Published:** 2023-12-13

**Authors:** Anthony J. Hannan

**Affiliations:** 1 Florey Institute of Neuroscience and Mental Health, University of Melbourne, Parkville, Victoria 3010, Australia; 2 Department of Anatomy and Physiology, University of Melbourne, Parkville, Victoria 3010, Australia

**Keywords:** human genome, repeatome, tandem repeat

## Abstract

Approximately half of the human genome includes repetitive sequences, and these DNA sequences (as well as their transcribed repetitive RNA and translated amino-acid repeat sequences) are known as the repeatome. Within this repeatome there are a couple of million tandem repeats, dispersed throughout the genome. These tandem repeats have been estimated to constitute ∼8% of the entire human genome. These tandem repeats can be located throughout exons, introns and intergenic regions, thus potentially affecting the structure and function of tandemly repetitive DNA, RNA and protein sequences. Over more than three decades, more than 60 monogenic human disorders have been found to be caused by tandem-repeat mutations. These monogenic tandem-repeat disorders include Huntington's disease, a variety of ataxias, amyotrophic lateral sclerosis and frontotemporal dementia, as well as many other neurodegenerative diseases. Furthermore, tandem-repeat disorders can include fragile X syndrome, related fragile X disorders, as well as other neurological and psychiatric disorders. However, these monogenic tandem-repeat disorders, which were discovered via their dominant or recessive modes of inheritance, may represent the ‘tip of the iceberg’ with respect to tandem-repeat contributions to human disorders. A previous proposal that tandem repeats may contribute to the ‘missing heritability’ of various common polygenic human disorders has recently been supported by a variety of new evidence. This includes genome-wide studies that associate tandem-repeat mutations with autism, schizophrenia, Parkinson's disease and various types of cancers. In this article, I will discuss how tandem-repeat mutations and polymorphisms could contribute to a wide range of common disorders, along with some of the many major challenges of tandem-repeat biology and medicine. Finally, I will discuss the potential of tandem repeats to be therapeutically targeted, so as to prevent and treat an expanding range of human disorders.

## Introduction

Our understanding of the human genome has been transformed in recent decades by genome sequencing and other approaches to mapping genetic mutations and polymorphisms. For monogenic disorders, this has involved identification of causative mutations. These mutations can take a variety of different forms, affecting both protein coding and non-coding sequences across the genome, and ranging from point mutations to structural mutations. As approximately half of the human genome is constituted by repetitive sequences, or repeatome [1], this provides a rich substrate for mutation, as many types of repetitive DNA, including tandem repeats, are highly mutatable. A small subset of the approximately two million tandem repeats distributed across the human genome have thus far been implicated in monogenic disorders, as discussed below. However, this may only represent a fraction of the total contribution of tandem repeats to human health and disease. Given the latest estimate that ∼8% of the entire human genome consists of tandem repeats [2], there is enormous scope for tandem repeats to affect human development, function and dysfunction.

For polygenic disorders, approaches such as genome-wide association studies (GWAS) have mapped genes and intergenic regions associated with a variety of human conditions. However, GWAS of human disorders and traits has left ‘missing heritability’ for these polygenic disorders [3,4]. The approximately two million tandem-repeat sequences (tandemly repeated DNA motifs) in the human genome have only recently become a target for genome-wide studies [5–9]. It has been proposed that tandem repeats can contribute to this missing heritability for a wide range of common disorders [3]. This article will focus predominantly on this issue, and its relevance to our understanding of the pathogenesis of a wide range of disorders, as well as ongoing challenges in the field of tandem repeats. The relevance to the prevention and treatment of disease will also be discussed.

## Mapping the landscape of tandem repeats in genomes from humans and other species

It is clear that the approximately two million tandem repeats (including short tandem repeats, or STRs, and variable number tandem repeats, or VNTRs) in the human genome are not unique to our bipedal primate species (which is only one of tens of millions of extant species that share the planet with us). Comparative genomics demonstrates high homologies of human genome-wide tandem repeats with other primates, other mammals and more disparate vertebrates [10]. Furthermore, invertebrate genomes are also extensively populated by tandem repeats, as are other non-animal species, from plants to microbes. This comparative genomics can help us understand how different types of tandem repeats, in different parts of genomes, evolved a diversity of structures and functions. These biological roles, at the molecular level, can include regulation of epigenetics, gene expression, RNA structure and function, and protein structure and function [11].

Another key source of tandem-repeat variability involves heterogeneity across humans, both healthy and diseased populations. Comparative human genomics reveals tight biological constraints on many thousands of tandem repeats. These constraints can be most extreme for tandem repeats that encode amino-acid repeats in proteins, such as polyglutamine tracts. One extreme example is the CAG/glutamine repeat in the FOXP2 gene/protein, which is ∼40 glutamines in length in the protein, and almost invariant in humans [12,13]. Notably, a family with a specific mutation (outside the tandem repeat) in the FOXP2 gene, had an extreme speech disorder [12], reflecting the strong evolutionary pressures on this gene during human evolution [13]. In contrast, the CAG/glutamine repeat in the *huntingtin* (*HTT*) gene/protein that expands to cause Huntington's disease (HD) is far more polymorphic in the general population, with evidence for functional impacts (short of the HD threshold) of this tandem-repeat polymorphism (TRP) in the non-HD general population [14–17]. In fact, a key characteristic of ‘pathogenic repeats’ (those associated with monogenic tandem-repeat disorders) is that they tend to be polymorphic in the human population, relative to other tandem repeats [2,18].

Comparative genomics at the DNA level will thus have much to offer with respect to understanding the evolution and function of the millions of tandem repeats, which are currently largely ‘genomic dark matter’. Furthermore, comparative genome-wide epigenomics, transcriptomics and proteomics (of tandemly repeated DNA, RNA and polypeptide sequences) will also be highly informative. Many tandem repeats located outside of coding regions appear to regulate epigenetic modifications, and thus the spatiotemporal control of gene expression [7,19,20]. Similarly, the majority of the human genome can be transcribed, and tandemly repeated RNA sequences can modulate RNA structure and function in many different ways [21–24]. Finally, tandemly repeated DNA sequences in coding regions, and sometimes outside of coding regions via repeat-associated non-ATG (RAN) translation [25–27], can encode repetitive amino-acid sequences within proteins, thus modulating various aspects of protein structure and function.

## Monogenic tandem-repeat disorders and tandem-repeat contributions to polygenic disorders

There are now well over 60 monogenic tandem-repeat disorders identified, including many neurological disorders, and many others that also impinge upon the nervous system. Furthermore, this list of tandem-repeat disorders continues to expand, year by year. As these tandem-repeat disorders have been extensively reviewed in recent years (e.g. [11,28–39]), their individual pathogenic mechanisms, biologies and therapeutic targets will not be a focus of this article.

These monogenic disorders include Huntington's disease, Friedreich ataxia, various spinocerebellar ataxis, fragile X syndrome, and C9ORF72-associated amyotrophic lateral sclerosis (motor neuron disease) and frontotemporal dementia. The large number of monogenic tandem-repeat disorders is direct evidence of the functional importance of these tandem repeats. It demonstrates that the human body, and the nervous system in particular, is intolerant of major mutations (particularly expansions) in many of these tandem repeats.

However, the greatest collective burden of disease in humans is not due to monogenic disorders, but rather is associated with more common polygenic disorders (and their complex and heterogenous pathogenic mixtures of genetic and environmental risk factors, or genomes and ‘enviromes’; [40]). The era of GWAS, which has thus far been largely based on microarrays genotyping single-nucleotide polymorphisms (SNPs), or single-nucleotide variants (SNVs), has found genetic associations across the genome for a wide variety of human disorders and traits. However, GWAS has also left substantial ‘missing heritability’, and it is an urgent priority to fully understand genetic contributions to disease, including those residing in the repeatome, and tandem repeats in particular.

It has been previously proposed that tandem repeats may make a major contribution to the missing heritability of polygenic disorders [3]. In recent years, genome-sequencing approaches, together with innovations in tandem-repeat bioinformatics, have provided new insights into common conditions such as autism [41–43], which have been followed up in subsequent studies [44]. There is evidence, although less extensive than for autism, that tandem-repeat mutations (and polymorphisms) could contribute to risk for schizophrenia [45,46] and Parkinson's disease [47]. Other genome-wide approaches to tandem repeats have revealed major contributions to various forms of cancer [48,49]. A recent study identified VNTRs with very strong associations to glaucoma and colorectal cancer [49]. Furthermore, genome-wide tandem repeats may not only contribute to common polygenic disorders, but also complex traits [50].

## Towards comprehensive mapping of tandem-repeat contributions to human disorders

Considering how few polygenic disorders have been thoroughly investigated with respect to tandem-repeat associations, there is enormous potential for discovery. In theory, existing GWAS datasets based on SNP-chip microarrays can be reanalysed with imputation approaches, so that tandem repeats linked to disease-associated SNPs can be imputed [6]. However, a problem with such approaches is that many tandem repeats mutate more frequently (are far more mutable) than single nucleotides. Therefore, SNPs are unlikely to ‘tag’ many tandem repeats, as the increased mutability of tandem repeats may confound such linkage approaches [3].

As the cost of genome sequencing has decreased, genome-sequencing approaches have continued to transform clinical genetics. Whole-genome sequencing (WGS) and whole-exome sequencing (WES; cheaper and faster, but less comprehensive) allows detailed mapping of tandem repeats throughout genomes and exomes, and comparison between disease and control populations, or across the phenotypic spectrum of different traits. One caveat is that genome/exome sequences generated from short-read sequencing (e.g. Illumina) may not be able to accurately capture longer tandem-repeat sequences [10,11]. However, more recent long-read sequencing technologies (e.g. Oxford Nanopore Technologies and PacBio) are better positioned to fully map tandem repeats, and their associations with human disorders [10,11,51,52]. Furthermore, long-read sequencing technology is improving variant detection and the identification of causal variants in human disease. For example, long-read sequencing is replacing Southern blots as gold-standard genotyping for many repeat-expansion diseases [52].

## Expanding technologies to explore comparative and clinical tandem-repeat genomics

The science of tandem repeats, like many other areas of science, has been catalysed by new technologies and analytic approaches. In the case of tandem-repeat sequencing, the advent of long-read sequencing technologies (as mentioned above) has opened up new possibilities regarding genome-wide mapping of tandem repeats, within and across individuals [10,11,35,52–55]. However, complementary advances have been made using novel bioinformatic approaches [8,29,56–73]. In addition to new bioinformatic tools to interrogate tandem repeats, there has also been progress with the development of systematic cataloging and databasing of tandem repeats (e.g. [74]).

The characterisation and mapping of tandem repeats across the human genome are thus becoming more routine (e.g. [5,75–77]). Importantly, genome-wide bioinformatic approaches have begun to link tandem-repeat sequences to molecular mechanisms, such as regulation of epigenetic modifications and associated gene expression [7,20,78,79]. However, there is an urgent need for further progress, so that whole-genome sequencing can be used routinely, and affordably, to accurately genotype all tandem repeats in the genome, and reliably identify pathogenic variants.

## Repeating themes informing therapeutic targets for tandem-repeat disorders

The ultimate aim of clinical genetics and genomics is to facilitate novel approaches to prevent, treat, and eventually cure, human disorders. The biology of tandem repeats provides a rich source of therapeutic targets. For example, in Huntington's disease (HD), a range of different approaches are being taken to either correct the tandem-repeat (CAG) expansion mutation (for example by CRISPR gene editing), lower somatic gene expression levels (for example by antisense oligonucleotide therapeutics; [80,81]), or target ‘downstream’ pathogenic pathways, such as polyglutamine toxicity (e.g. [31,82,83]) and various aspects of brain-body interactions, including the microbiota-gut-brain axis (e.g. [84,85]).

CRISPR (clustered regularly interspaced short palindromic repeats) gene editing is being pursued by several academic groups and companies, as are antisense oligonucleotides [86] and small-molecule drugs [87]. A specific variant of the CRISPR approach has been developed to target transcription from tandem repeats [88] and these and other approaches offer significant hope for the prevention and treatment of such fatal monogenic diseases.

In theory, somatic gene-editing approaches, such as those provided by CRISPR technologies, could be applied to all tandem-repeat disorders. In practice, there are many remaining challenges. One challenge is delivery, and this is a problem that has faced the gene therapy field more widely, for decades. The brain is a particularly challenging organ for targeted gene editing, due primarily to the blood-brain barrier. Another challenge is constituted by potential off-target effects of gene editing. This is a particularly significant problem for tandem-repeat disorders, as the repetitive nature of the target can be a challenge for specificity. Similarly for the many autosomal dominant tandem-repeat disorders (with Huntington's disease being amongst the most common), ideal therapeutic approaches will be allele specific, and thus the therapy must selectively target the tandem repeat-expanded allele, leaving the normal ‘healthy’ allele intact [81,86].

For some tandem-repeat disorders, it appears that somatic expansion of the tandem repeat contributes to pathogenesis (e.g. [89–93]). This may occur during development and/or adulthood, via various mechanisms (e.g. [94,95]). Therefore, the tandem-repeat target for gene-editing may vary in length between different cells, systems and organs. This presents an additional challenge for gene-editing approaches that target highly specific repeat lengths. One approach to target such somatic repeat expansion involves small-molecule therapeutics that could inhibit expansion, and perhaps even induce contraction of pathologically expanded repeats (e.g. [96–98]).

## Future directions and grand challenges

The first grand challenge of tandem-repeat biology will be accurately mapping all tandem repeats across all known species. We know for example that tandem repeats have evolved extensively during human evolution, in large populations (e.g. [99]), with short-term mutational dynamics (without long-term evolutionary pressures) also observed in individual families (e.g. [100]). We need to know much more about how tandem repeats evolve (e.g. [22,101]) and how this relates to organismal development, structure, function and evolution.

The second grand challenge will be to fully characterise tandem-repeat polymorphisms across large human populations, and relate this to phenotypic information. This approach is beginning to emerge (e.g. [102]). However, with approximately eight billion humans on the planet, many of whom are located in countries embarking on population-wide genome sequencing approaches to facilitate genomic and precision medicine, we are only at the beginning of a long journey. Integrating this tandem-repeat genomics with phenomics, to establish how tandem-repeat polymorphisms and mutations contributed to human diseases and traits, will facilitate novel approaches to the prevention and treatment of a wide variety of disorders.

The third grand challenge is to accelerate therapeutic development (discussed above) to find novel ways to prevent or treat all human disorders involving tandem repeats. The development of new therapies will in some cases be tandem repeat, and disease, specific. However, commonalities across disorders (e.g. polyglutamine and polyalanine tracts), may inform novel approaches targeting multiple diseases. Furthermore, tandem-repeat therapeutics may have DNA, RNA or protein targets, and involve a wide range of rapidly evolving technologies, from small molecules, to DNA and RNA editing, and biologics targeting repetitive RNA and amino-acid sequences.

## Conclusions

Due to space constraints, this article has only been able provide a flavour of the excitement and enormous potential of the tandem-repeat field. Whilst the focus has been on ‘tandem-repeat medicine’ and ‘tandem-repeat therapeutics’, the potential applications run far beyond human biology and disease. As the vast majority, if not all, other species have tandem repeats in their genomes, our understanding of genome-wide tandem-repeat biology at molecular, cellular and systems levels could have immense impacts. Whilst tandem repeats, and the rest of the repeatome, have long been the ‘dark matter of the genome’, scientific illumination promises to be transformative. There are undoubtedly novel applications of tandem-repeat biology in ecology, conservation, agriculture, and beyond. But most excitingly, understanding and targeting tandem repeats offers new hope to prevent, treat, and eventually cure, a wide range of devastating disorders, thus improving human health and reducing morbidity and mortality.

## Summary

- Approximately 8% of the human genome consists of tandemly repeated DNA sequences, known as tandem repeats, located throughout both genic and intergenic regions
- These tandem repeats, the most common of which are short tandem repeats (STRs), have been associated with over 60 monogenic human disorders (e.g. Huntington's disease, many ataxias, amyotrophic lateral sclerosis, frontotemporal dementia, fragile X syndrome and other predominantly neurological disorders)
- Tandem repeats have recently been associated with common polygenic disorders, including autism, schizophrenia, Parkinson's disease, and many cancers, suggesting major involvement in ‘missing heritability’
- Tandem repeats constitute major therapeutic targets for this large, and expanding, list of human disorders, and current candidate approaches include tandem-repeat targeted CRISPR gene editing, antisense oligonucleotides, biologics and small-molecule drugs

## Open Access

Open access for this article was enabled by the participation of University of Melbourne in an all-inclusive *Read & Publish* agreement with Portland Press and the Biochemical Society under a transformative agreement with CAUL.

## Abbreviations

CRISPR: clustered regularly interspaced short palindromic repeats
GWAS: genome-wide association studies
HD: Huntington's disease
HTT: huntingtin
SNPs: single-nucleotide polymorphisms
SNVs: single nucleotide variants
STRs: short tandem repeats
VNTRs: variable number tandem repeats
WES: whole-exome sequencing
WGS: whole-genome sequencing
